# Supplementary material for: Imaging and writing magnetic domains in the non-collinear antiferromagnet Mn3Sn
Source: Nat Commun. 2019 Nov 29;10:5459. doi: 10.1038/s41467-019-13391-z (PMC6884521; doi:10.1038/s41467-019-13391-z)
Supplement: Supplementary file 1 — Supplementary Information [file 41467_2019_13391_MOESM1_ESM.pdf]

Supplementary information: Imaging and writing magnetic domains in the non-collinear antiferromagnet Mn_3Sn

Helena Reichlova,[1] Tomas Janda,[2] Joao Godinho,[2,3] Anastasios Markou,[4] Dominik Kriegner,[3,4] Richard Schlitz,[1] Jakub Zelezny,[3] Zbynek Soban,[3] Mauricio Bejarano,[5] Helmut Schultheiss,[5] Petr Nemec,[2] Tomas Jungwirth,[3,6] Claudia Felser,[4] Joerg Wunderlich,[3,7] and Sebastian T. B. Goennenwein[1]

[1]*Institut für Festkörper- und Materialphysik and Würzburg-Dresden Cluster of Excellence ct.qmat, Technische Universität Dresden, 01062 Dresden, Germany**

[2]*Faculty of Mathematics and Physics, Charles University, Ke Karlovu 3, 121 16 Prague 2, Czech Republic*

[3]*Institute of Physics ASCR, v.v.i., Cukrovarnická 10, 162 53, Praha 6, Czech Republic*

[4]*Max Planck Institute for Chemical Physics of Solids, Nöthnitzer Straße 40, 01187 Dresden, Germany*

[5]*Helmholtz-Zentrum Dresden-Rossendorf, Institute of Ion Beam Physics and Materials Research, Bautzner Landstraße 400, 01328 Dresden, Germany*

[6]*School of Physics and Astronomy, University Of Nottingham, NG7 2RD, Nottingham, United Kingdom*

[7]*Hitachi Cambridge Laboratory, Cambridge CB3 0HE, United Kingdom*

* Corresponding author: helena.reichlova@tu-dresden.de

SUPPLEMENTARY NOTE 1

Magneto-optical Kerr effect (MOKE) was sucessfully used to probe the surface magnetic character of Mn_3Sn single crystal (Fig. 4 in Ref.[4]). There is, however, several important differences between the MOKE and STGM which are discussed in the following.

MOKE is surface sensitive; the probing depth is limited by the penetration depth of the light into the material and in case of a metal is typically ∼20 nm. For example in Mn the light intensity decreases to 1/e in the depth 16 nm in Sn 10 nm and in Fe 19 nm [3, 6], assuming reflection and interference on interfaces is neglected. The STGM technique relies on the thermal gradient in direction perpendicular to the sample plane (we assume the in-plane gradients cancel due to the radial symmetry). The sample holder (or the substrate in case of thin films) serves as the heat sink for the laser generated heat gradient and therefore the probing depth of the STGM is not only surface sensitive, but can penetrate several 100 nm or even several microns into the material. From a thin film perspective, the STGM technique thus is rather bulk sensitive. The surface sensitivity is a limiting factor especially for thin films which are often prepared in multilayered stacks (either with a capping layer or as a functional bilayer with a heavy metal or ferromagnets). The STGM can probe also buried layers below several nanometers of Pt or another heavy metal that are basically invisible to MOKE

MOKE is spectrally dependent; MOKE is a very useful to probe (surface) magnetism in one sample; however, comparing multiple samples can be difficult. The spectral dependency of MOKE can be different not only for different materials but importantly also for different thickness of the same material due to varying interference on multilayers. Therefore MOKE might not be suitable to, for example, compare domain size in films with varying thickness. STGM allows for direct comparison of the magneto-thermal response (similar to comparing anomalous Hall conductivity of various materials) and it is obviously not sensitive to interference. The MOKE spectral dependency also implies that the experimental setup is significantly more complex and materials with weak MOKE coefficient might be invisible to the MOKE technique completely. Moreover, in antiferromagnets the MOKE spectral dependency is generally hard to measure if the manipulation of the antiferromagnetic order is not readily available.

Geometry of MOKE and STGM experiment. The thin epitaxial films can be grown with

the [0001] axis out of the sample plane. Ref.[4] takes advantage of the bulk single crystal sample, therefore the authors can shine the light from any side to see Polar MOKE. In Ref.[4] light is applied along [2-1-10] direction, it is not obvious that any signal could be measured if light would be applied along [0001] and therefore if the MOKE would be applicable to the epitaxial thin films at all.

SUPPLEMENTARY NOTE 2

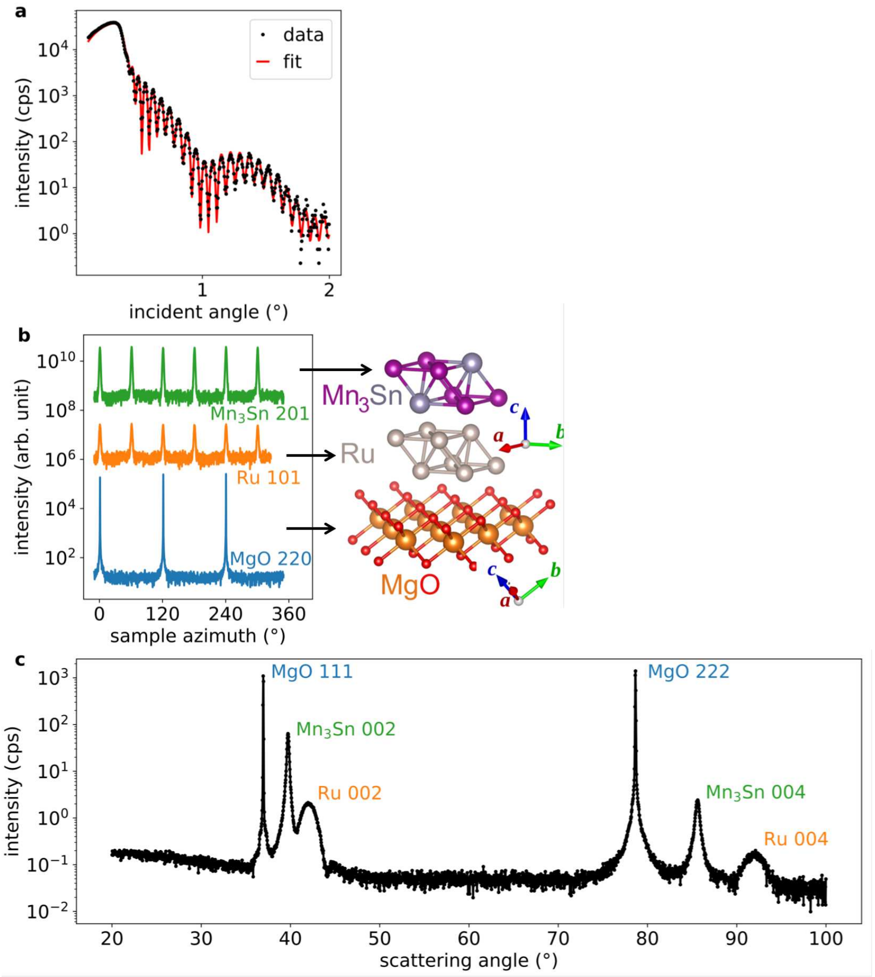

Supplementary Figure 1. Thin film characterization by X-ray reflectivity and X-ray diffraction. (a) X-ray reflectivity data and fit of the MgO/Ru(3 nm)/Mn_3Sn(50 nm) stack showing that the films are smooth and the surface and interface roughness is below 1 nm. (b) X-ray diffraction azimuth scans probing the inplane epitaxial relationship. Shown is the diffraction signal from the 220, 101, and 201 Bragg peaks of the MgO substrate, the Ru as well as Mn_3Sn layers, respectively. (c) X-ray diffraction radial scan proving the epitaxial growth of the Ru and Mn_3Sn layers on the single crystalline MgO (111) substrate. For the Ru and Mn_3Sn exclusively the (00L) family of Bragg peaks is detected. Therefore we conclude that the epitaxial relationship is (111)[1-10]MgO ‖(001)[100] Ru‖(001)[100]Mn_3Sn. The corresponding unit cell arrangement of the three layers MgO(red-orange)/Ru(grey)/Mn3Sn(purple) is shown next to panel (b).

The Mn_3Sn films were grown on MgO(111) single crystal substrates with a thin Ru buffer layer. The unit cell orientations shown in Fig. 1 illustrate that the three material systems fit well on top of each other. The films were characterized by X-ray reflectivity and X-ray diffraction measurements. The results are summarized in Fig. 1. The results show that the films are smooth and the surface roughness is below 1 nm. The diffraction confirms the epitaxial growth of the Mn_3Sn films.

SUPPLEMENTARY NOTE 3

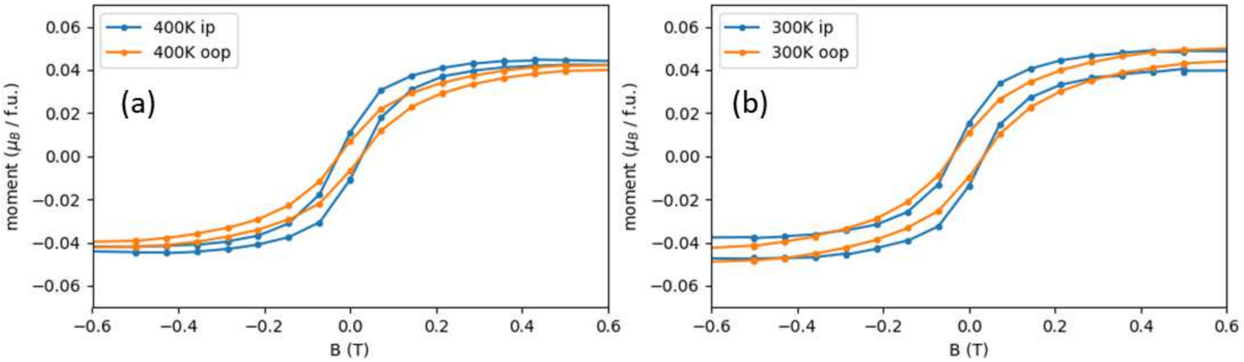

Supplementary Figure 2. Magnetometry. A typical magnetometry data measured at 400 K (a) and 300 K (b) with magnetic field in the sample plane (ip) and out of the sample plane (oop). A small net moment is observed, which, however, does not vary between 300 K and 400 K and, therefore, a ferromagnetic-like impurity can be excluded as a source of the thermo-voltage at 400 K.

The magnetometry data measured using a typical sample (50 nm) are shown in Fig. 2 for two temperatures. A small net moment is observed, which, however, has the same amplitude at 300 K and 400 K. Therefore the V_{ANE} measurements at 400 K discussed in the main text cannot be explained by a ferromagnetic impurity phase. We also note that compared to bulk Mn_3Sn, the thin film magnetometry data might reflect not only the net magnetic moment but also small contribution from interfaces or grain boundaries and most importantly a spurious signal from the MgO substrate. This might also explain why the magnetic moment per Mn atom (0.01 μ_B/Mn) which we find in our thin films is larger than for bulk Mn_3Sn,

similar to another thin film study [4]. In our thin films we do not observe evidence for the formation of a spin glass below 50 K, as reported in bulk Mn_3Sn [5].

The spin glass formation in kagome antiferromagnets is explained by a model of spin folds in several reports [2, 10]. They consider the in-plane anisotropy responsible for spin glass formation since a spin reorientation into the out-of-plane direction is energetically more demanding. A similar mechanism could also result in the absence of a spin glass phase in our Mn_3Sn thin films. We observe larger coercive (reversal) field in thin films compared to the bulk Mn_3Sn. This can be, of course, due to a larger anisotropy, but it can also be due to stronger pinning at local defects, grain boundaries, dislocations, local strain variations and magnetostrictions. Detailed studies is beyond the scope of this manuscript and represents an important future task.

SUPPLEMENTARY NOTE 4

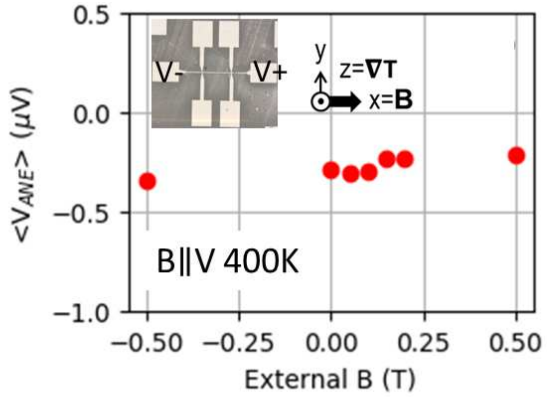

Supplementary Figure 3. Thermo-voltage detected along the direction of the magnetic field. When the global hysteresis curve is measured along the magnetic field (see the inset for the experimental geometry) no variation of the measured voltage is observed. It confirms the anomalous Nernst geometry as discussed in the main text.

To study the anisotropy of the ANE we changed the geometry of the experiment in the way that we detect the thermovoltage along the applied magnetic field as shown in the inset of Fig.3. In the magnetic reversal region the sample can in principle break into multiple domains. Domains with **g** parallel to the x-direction do not contribute to the measured voltage V_{ANE} and these domains represent the majority after the field was applied along the

x-direction. The minority of the remaining domains have non-zero projection of **g** to the y-direction, but could, however, be averaged out due to their random distribution. This can be seen in Fig. 3, where the global hysteresis curve measured at contacts along the magnetic field direction exhibits no variation with the magnetic field. The uniform constant background might originate in a thermal counterpart of ordinary magnetoresistance or parts of the sample are not possible to be polarized even at 400 K.

SUPPLEMENTARY NOTE 5

The resolution of the STGM is given by the size of the laser spot [11]. In our case the laser beam has a Gaussian intensity profilesith FWHM=1.5 μm. After magnetically polarizing the sample at 400 K (Fig. 2c,d in the main text) the large amplitude of the anomalous Nernst coefficient would suggest that the sample forms large domains and the measured signal reflects one orientation of the **g** vector. When the sample, however, breaks into multiple domains the laser spot size is very likely larger than the domain size and the resulting thermo-voltage is an average of multiple domains. This explains the relatively continuous variation of the thermo-voltage amplitude for example in Fig. 2a,b of the main text. The resolution of the STGM can be obviously pushed down by reducing the laser spot size or by advanced modeling of the measured signal by considering the profile of the laser beam and its convolution with the magnetic structure [1]. A dramatic improvement of the resolution would, however, be achieved by employing a more localised source of thermal gradient, for example a heated atomic force microscopy tip [7] or near field scanning optical mircoscopy [8]. These techniques would yield smaller amplitude of the thermo-voltage, however, considering the large response ($\sim 1\ \mu V$) observed in our experiments, the signal should be still possible to detect.

SUPPLEMENTARY NOTE 6

At 375 K a weak variation of the V_{ANE} pattern with the application of 0.5 T magnetic field can be observed as seen in Fig. 4. We interpret this as an evidence that the domains with weakest anisotropy can be reoriented while the majority of the domains still remains insensitive to the magnetic field.

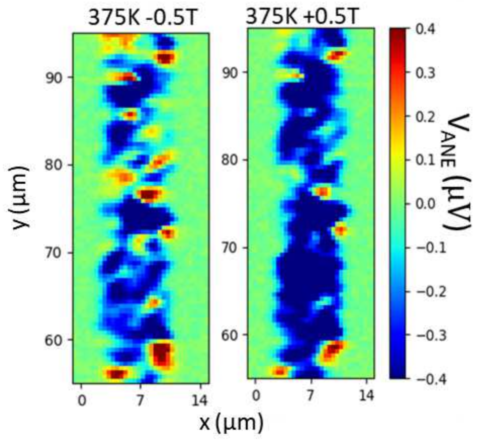

Supplementary Figure 4. STGM scans as a function of magnetic field at 375 K. A weak variation of the V_{ANE} pattern can be seen corresponding to the reversal of domains with weakest anisotropy.

SUPPLEMENTARY NOTE 7

At 400 K the majority of magnetic domains can be reversed by application of 0.5 T magnetic field in the sample plane. In our experiments, the magnetic field was applied perpendicular to the voltage detection direction and changed in steps in the range of +/-0.5 T. In each step with the given fixed magnetic field applied a full STGM map of the scanned area was recorded. The individual maps for all magnetic field values studied are shown in Fig. 5, the corresponding $<V_{ANE}>$ is discussed in the main text (Fig. 2e). The clear remanence and hysteresis can be seen from the two maps recorded at 0 T magnetic field after +0.5 T (Fig. 5(c)) and -0.5 T (Fig. 5(j)) was applied, respectively. Note that in the reversal region (between -150 mT and and +150 mT) multiple domains are formed and the reorientation is not uniformly changing color from blue to red.

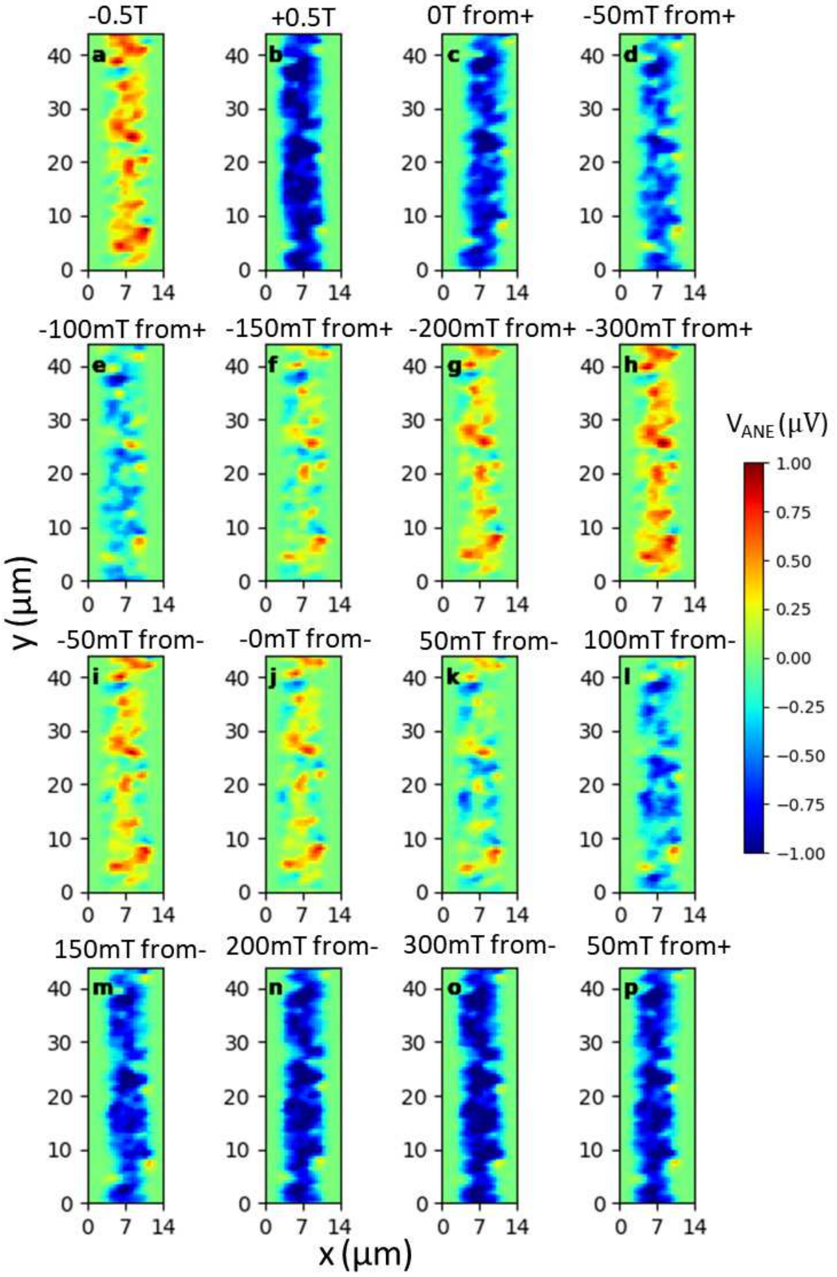

Supplementary Figure 5. STGM scans as a function of magnetic field at 400 K. The magnetic field was changed in steps in the order from (a) to (p) and for each field value a STGM map was recorded. The corresponding $\langle V_{ANE} \rangle$ is discussed in the main text (Fig. 2e). Between the measurements (h) and (i) a field of -0.5 T was applied and between (o) and (p) a field of +0.5 T was applied. The text above individual maps indicates the applied magnetic field and the history of magnetic field. The color bar shows the scale of measured voltage.

SUPPLEMENTARY NOTE 8

To study the impact of the temperature on the V_{ANE} signal the sample is first cooled to 17 K (the lowest experimentally available temperature) in zero field and a STGM map is recorded. The sample temperature is then increased in steps and at each studied temperature a STGM map is recorded as shown in Fig. 6. At certain temperatures, the temperature induced stresses in the setup result in a small spatial drift and therefore the image of the Hall bar is slightly deformed.

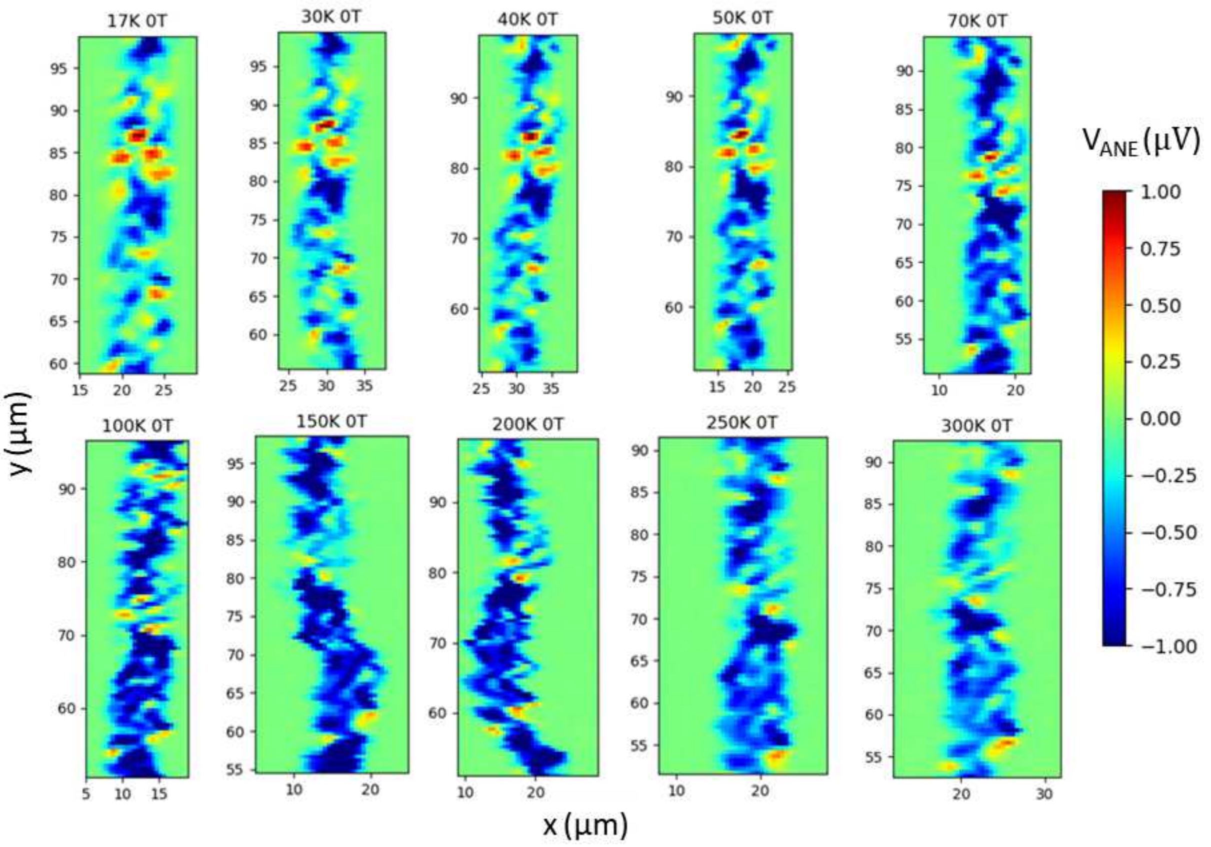

Supplementary Figure 6. Temperature dependency of the STGM maps. After cooling the sample in zero magnetic field to 17 K, consequently the temperature is raised in steps and at each temperature a STGM map is recorded. No magnetic field is applied during this experiment. The color bar shows the scale of measured voltage.

SUPPLEMENTARY NOTE 9

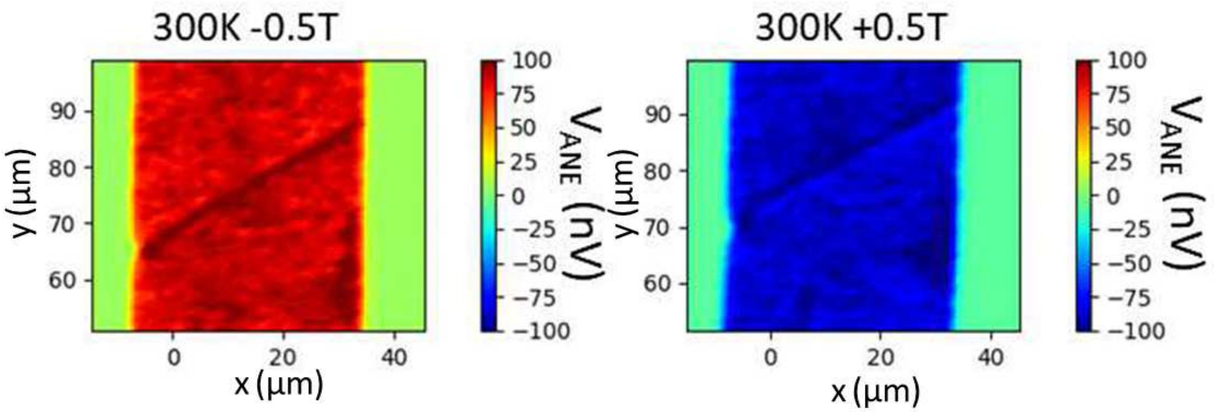

Supplementary Figure 7. Calibration of ∇T against Co_2MnGa thin film. The ferromagnetic Co_2MnGa sample was measured under identical conditions when magnetic field of +0.5 T and -0.5 T was applied. This magnetic field is sufficient to reorient the magnetization in the film plane. The color bar shows the scale of measured voltage.

The thermal gradient generated by the laser spot was calibrated against a Co_2MnGa thin film on a MgO substrate. The sample was patterned into a Hall bar and measured under exactly the same conditions as the Mn_3Sn films. The sample was polarized by +0.5 T and -0.5 T as can be seen in Fig. 7 and from these scans the amplitude of the anomalous Nernst voltage was extracted. Because the anomalous Nernst coefficient in our Co_2MnGa is known to be $2\,\mu V/K$ [9], the thermal gradient can be evaluated. At 300 K and 10 mW laser power we estimate $\nabla T \sim 2\,K/\mu m$, which is in good agreement with other laser induced thermal gradients [11]. Using this value of the laser generated thermal gradient also for our Mn_3Sn data, we can quantify the anomalous Nernst coefficient. A different size of the Co_2MnGa Hall bar and Mn_3Sn Hall bar was corrected by considering a simple model of parallel resistances as described for example in the Supplementary Information of the paper by Weiler et al.[11].

SUPPLEMENTARY NOTE 10

All heat assisted domain writing experiments were performed at 300 K with a reading (STGM scanning) laser power of 10 mW. All written domain patterns remain insensitive to

the magnetic field 0.5 T, as can be seen in Fig. 8.

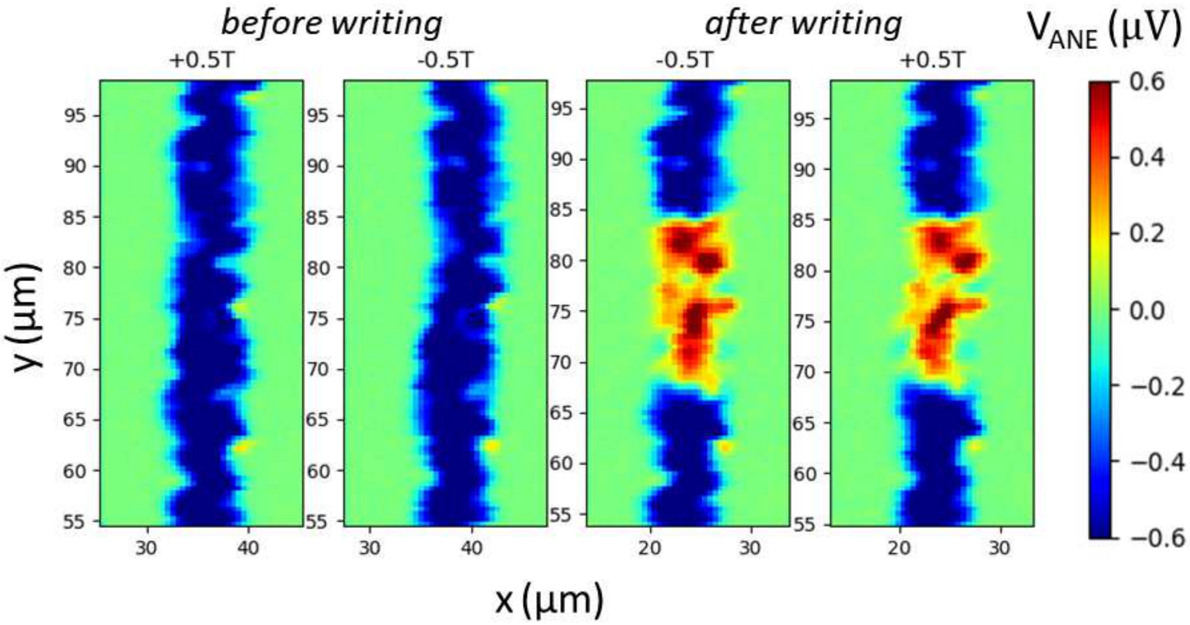

Supplementary Figure 8. Robustness of the magnetic structure at 300 K. At 300 K and with the laser power of 10 mW the magnetic field cannot alter the magnetic state of the sample. Magnetic field +0.5 T and -0.5 T was applied before the domain writing and after the domain writing, in both cases the written pattern was unchanged. The color bar shows the scale of measured voltage.

To test the impact of the magnetic fields at 300 K even further, we placed the sample into a different cryostat in which a magnetic field of 6 T was applied in the sample plane. Afterwards the sample was again inserted into the optical setup and the STGM was performed. The domain pattern remains unchanged even by the application of 6 T as can be seen in Fig. 9. This behavior is different than for a case of a ferromagnet.

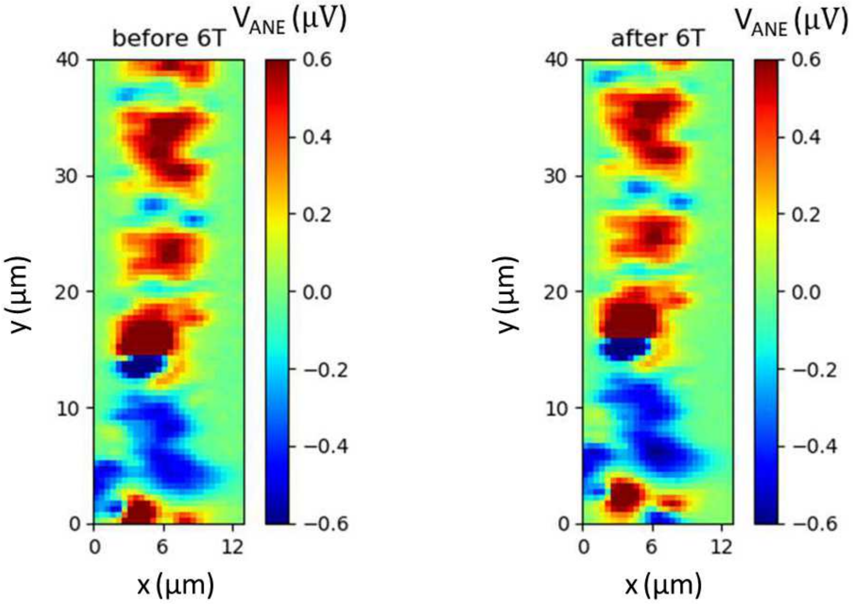

Supplementary Figure 9. Robustness of the magnetic structure at 300 K. At 300 K 6T cannot erase the magnetic domains written to the sample. The written pattern remains unchanged before and after 6T is applied to the sample. The color bar shows the scale of measured voltage.

---

[1] Bhallamudi, V. P., Wolfe, C. S., Amin, V. P., Labanowski, D. E., Berger, A. J., Stroud, D., Sinova, J., and Hammel, P. C. (2013). Experimental demonstration of scanned spin-precession microscopy. *Physical Review Letters*, 111(11).

[2] Bisson, W. G. and Wills, A. S. (2008). Anisotropy-driven spin glass transition in the kagome antiferromagnet hydronium jarosite, (h3o)fe3(SO4)2(OH)6. *Journal of Physics: Condensed Matter*, 20(45):452204.

[3] Golovashkin, A. and Motulevich, G. (1964). Optical and electrical properties of tin. *Journal of Experimental and Theoretical Physics*, 19(2):310.

[4] Higo, T., Qu, D., Li, Y., Chien, C. L., Otani, Y., and Nakatsuji, S. (2018). Anomalous hall effect in thin films of the weyl antiferromagnet mn3sn. *Applied Physics Letters*, 113(20):202402.

[5] Ikhlas, M., Tomita, T., Koretsune, T., Suzuki, M.-T., Nishio-Hamane, D., Arita, R., Otani, Y., and Nakatsuji, S. (2017). Large anomalous nernst effect at room temperature in a chiral antiferromagnet. *Nature Physics*, 13(11):1085–1090.

[6] Johnson, P. and Christy, R. (1974). Optical constants of transition metals: Ti, v, cr, mn, fe, co, ni, and pd. *Physical Review B*, 9(12):5056–5070.

[7] Milner, A. A., Zhang, K., Garmider, V., and Prior, Y. (2010). Heating of an atomic force microscope tip by femtosecond laser pulses. *Applied Physics A*, 99(1):1–8.

[8] Patoka, P., Ulrich, G., Nguyen, A. E., Bartels, L., Dowben, P. A., Turkowski, V., Rahman, T. S., Hermann, P., Kästner, B., Hoehl, A., Ulm, G., and Rühl, E. (2016). Nanoscale plasmonic phenomena in CVD-grown MoS_2 monolayer revealed by ultra-broadband synchrotron radiation based nano-FTIR spectroscopy and near-field microscopy. *Optics Express*, 24(2):1154.

[9] Reichlova, H., Schlitz, R., Beckert, S., Swekis, P., Markou, A., Chen, Y.-C., Kriegner, D., Fabretti, S., Park, G. H., Niemann, A., Sudheendra, S., Thomas, A., Nielsch, K., Felser, C., and Goennenwein, S. T. B. (2018). Large anomalous nernst effect in thin films of the weyl semimetal co2mnga. *Applied Physics Letters*, 113(21):212405.

[10] Ritchey, I., Chandra, P., and Coleman, P. (1993). Spin folding in the two-dimensional heisenbergkagoméantiferromagnet. *Physical Review B*, 47(22):15342–15345.

[11] Weiler, M., Althammer, M., Czeschka, F. D., Huebl, H., Wagner, M. S., Opel, M., Imort, I.-M., Reiss, G., Thomas, A., Gross, R., and Goennenwein, S. T. B. (2012). Local charge and spin currents in magnetothermal landscapes. *Physical Review Letters*, 108(10):106602.
