# Supplementary material for: Imaging and writing magnetic domains in the non-collinear antiferromagnet Mn3Sn
Source: Nat Commun. 2019 Nov 29;10:5459. doi: 10.1038/s41467-019-13391-z (PMC6884521; doi:10.1038/s41467-019-13391-z)
Supplement: Supplementary file 2 — Peer Review File [file 41467_2019_13391_MOESM2_ESM.pdf]

Editorial Note: This manuscript has been previously reviewed at another journal that is not operating a transparent peer review scheme. This document only contains reviewer comments and rebuttal letters for versions considered at *Nature Communications.*

REVIEWERS' COMMENTS:

Reviewer #1 (Remarks to the Author):

The authors revised the manuscript carefully according to the review report of the first round. With the newly added discussion and reference citation, I think the paper meets the standard of Nature Communications, and I recommend it to be published in the present form.

Reviewer #2 (Remarks to the Author):

As mentioned in my previous report, the work is nicely written, interesting and scientifically sound. Although it lacked the impact of a paper suitable for Nature Physics, I suggested the authors to transfer their work to Nature Communications. The second referee has the same opinion. Furthermore, the authors have included all recommendations by the referees.

I therefore recommend publication to Nature Communications.

Response Letter NCOMMS-19-29116-T

REVIEWERS' COMMENTS:

Reviewer #1 (Remarks to the Author):

The authors revised the manuscript carefully according to the review report of the first round. With the newly added discussion and reference citation, I think the paper meets the standard of Nature Communications, and I recommend it to be published in the present form.

Reviewer #2 (Remarks to the Author):

As mentioned in my previous report, the work is nicely written, interesting and scientifically sound. Although it lacked the impact of a paper suitable for Nature Physics, I suggested the authors to transfer their work to Nature Communications. The second referee has the same opinion. Furthermore, the authors have included all recommendations by the referees.

I therefore recommend publication to Nature Communications

We would like to thank the referees for the positive feedback and their contribution to improve the manuscript.
